# Surgical outcomes of subtotal colectomy with antiperistaltic caecorectal anastomosis vs total colectomy with ileorectal anastomosis for intractable slow-transit constipation

**DOI:** 10.1093/gastro/goz014

**Published:** 2019-05-12

**Authors:** Xiao-Yu Xie, Kong-Liang Sun, Wen-Hao Chen, Yan Zhou, Bao-Xiang Chen, Zhao Ding, Xue-Qiao Yu, Yun-Hua Wu, Qun Qian, Cong-Qing Jiang, Wei-Cheng Liu

**Affiliations:** 1 Department of Colorectal and Anal Surgery, Zhongnan Hospital of Wuhan University, Wuhan, Hubei, P. R. China; 2 Clinical Center of Intestinal and Colorectal Diseases of Hubei Province, Wuhan, Hubei, P. R. China; 3 Key Laboratory of Intestinal and Colorectal Diseases of Hubei Province, Wuhan, Hubei, P. R. China; 4 Colorectal and Anal Disease Research Center, Medical School of Wuhan University, Wuhan, Hubei, P. R. China; 5 Quality Control Center of Colorectal and Anal Surgery, Health Commission of Hubei Province, Wuhan, Hubei, P. R. China

**Keywords:** slow-transit constipation, colectomy, ileorectal anastomosis, antiperistaltic caecorectal anastomosis

## Abstract

**Background:**

Few studies have compared the surgical outcomes of different surgical procedures currently used to treat refractory colonic slow-transit constipation (STC), despite the increase in the number of cases. This study aimed to analyse the long-term surgical outcomes of subtotal colectomy with antiperistaltic caecorectal anastomosis (SC-ACRA) vs total colectomy with ileorectal anastomosis (TC-IRA) for severe STC.

**Methods:**

Between January 2005 and January 2015, we retrospectively collected clinical data of 55 patients who underwent TC-IRA (*n *=* *35) or SC-ACRA (*n *=* *20) for severe STC at our institution. The post-operative functional outcomes between the two groups were compared.

**Results:**

There were no significant differences in age (*P *=* *0.655), sex (*P *=* *0.234), period of constipation (*P *=* *0.105) and defecation frequency (*P *=* *0.698) between the TC-IRA and SC-ACRA groups. During a median follow-up period of 72 months (range, 12–120 months), there were no significant differences between the TC-IRA and SC-ACRA groups regarding the median number of bowel movements per day [3 (1/6–7) vs 3 (1/6–5), *P *=* *0.578], Cleveland Clinic Florida Constipation Score [2 (0–20) vs 2 (0–19), *P *=* *0.454], Cleveland Clinic Incontinence Score [0 (0–5) vs 0 (0–2), *P *=* *0.333] and Gastrointestinal Quality of Life Index [122 (81–132) vs 120 (80–132), *P *=* *0.661]. Moreover, there was no significant difference in the incidence of post-operative complications between the two groups (37.1% vs 25.0%, *P *=* *0.285).

**Conclusions:**

Our findings indicate that both TC-IRA and SC-ACRA are effective treatments for severe STC, with similar long-term outcomes.

## Introduction

Colonicslow-transit constipation (STC) is defined as reduced colonic motility and stool frequency, usually accompanied by abdominal bloating; it is one of the most common types of constipation encountered by colorectal surgeons [1]. Currently, it is estimated that 2%–28% of the Western population [2, 3] and 11.6%–14.3% of the Asian population have constipation [4, 5]. Of these, STC affects 13%–37% of patients with constipation and notably impacts their quality of life [1, 2, 6–8].

Although more conservative approaches have recently been reported, ablative procedures continue to play effective roles in treating STC [9–11]. Many STC patients still experience an infrequent urge to defecate, in addition to various degrees of abdominal pain or discomfort after conservative management, which causes laxative abuse in STC patients. Thus, surgery is recommended for patients with intractable symptoms of constipation who do not respond to medical management [12].

Sinceits first use in 1908, total colectomy with ileorectal anastomosis (TC-IRA) has become the most widely adopted procedure for the treatment of STC, with the effective relief of constipation and a reduction of its post-operative recurrence. Although most patients who undergo this procedure experience improved constipation symptoms, there is also an increased incidence of post-operative faecal incontinence, intestinal obstruction, diarrhoea and abdominal pain that reduces the patients’ post-operative quality of life [2, 9]. Therefore, several surgical alternatives for STC have been described, such as side-to-side caecorectal anastomosis and antiperistaltic caecorectal anastomosis with subtotal colectomy [2, 9, 13–15]. The advantages and disadvantages of these procedures are difficult to assess and the exact efficacy of these procedures compared with TC-IRA is unclear. For example, subtotal colectomy with end-to-end antiperistaltic caecorectal anastomosis, first reported in 2001 by Sarli *et al.* [15], seems to be a valid alternative to TC-IRA, with the advantages of potentially less functional impairment, superior structural function and better post-operative outcomes, while also having possible disadvantages such as an increased incidence of post-operative recurrence [13–15]. However, the cases reported were small and, with the few published studies that have compared the clinical outcomes of TC-IRA and subtotal colectomy with antiperistaltic caecorectal anastomosis (SC-ACRA), it is difficult to assess the relative merits of the two procedures. Thus, this study aimed to retrospectively evaluate and compare the long-term outcomes of patients with severe STC who underwent TC-IRA and SC-ACRA over the same period of time.

## Patients and methods

### Study design

Weconducted a retrospective cohort study and identified all patients who underwent SC-ACRA or TC-IRA in Zhongnan Hospital of Wuhan University. Both procedures were performed by the same team of experienced colorectal surgeons. All patients who underwent abdominal surgery for treating STC at our centre between January 2005 and January 2015 were considered for this clinical research. This study was approved by the Clinical Trial Ethics Committee of Zhongnan Hospital of Wuhan University and was registered in the Chinese Clinical Trial Registry (Registration number: ChiCTR-ORB-15007316). All the procedures adhered strictly to the Declaration of Helsinki. We collected all the patients’ telephone numbers and made an appointment with each participant for an interview at the outpatient clinics of Zhongnan Hospital, Wuhan University. Written informed consent was obtained from the participants prior to the study.

### Patients’ selection

Between January 2005 and January 2015, 78 patients with severe STC underwent abdominal surgery at our centre. Twenty-three patients were excluded because of an abdominal total colonic excision (*n *=* *13), previous abdominal surgery (*n *=* *5) or previous transanal surgery (*n *=* *5). Ultimately, 20 patients who underwent SC-ACRA and 35 patients who underwent TC-IRA were included in the present study.

All participants underwent clinical assessments for STC before surgery, according to the Rome III criteria. All participants had reduced colonic motility and decreased stool frequency, which failed to improve after the non-operative management of constipation including dietary modification with fibre and fluid supplementation and the use of osmotic laxatives. All participants underwent diagnostic examinations before surgery as follows: (i) repeated gastrointestinal transit time (GITT) study using 20 radiopaque markers; (ii) barium enema and colonoscopy; (iii) defecography or simultaneous pelvicography and colpocystodefecography, including pelvicography, vaginal opacification, voiding cystography and defecography; (iv) anorectal manometry; and (v) endorectal ultrasonography. GITT was measured by plain abdominal radiography on the 6th, 24th, 48th and 72nd hours. If more than 20% of the markers were not eliminated after 3 days, the test was considered positive for STC. Additional radiography examinations were performed for another 2 days if the test was positive, which allowed a differentiation between total and segmental STC. Small-bowel dysmotility was defined as the retention of two or more markers in the ileum after 6 hours. Further assessment included standardized questionnaires for Cleveland Clinic Florida Constipation Scores (CCFIS) [16], Gastrointestinal Quality of Life Index (GIQLI) [17] and Cleveland Clinic Incontinence Scores (CCIS) [18]. The questionnaires were completed before operation by four authorized surgeons.

The indications for SC-ACRA were similar to those for TC-IRA and were based on the Rome III criteria: (i) a history of severe refractory STC for at least 5 years; (ii) a stool frequency of fewer than two per week; (iii) unresponsiveness to appropriate and intensive medical treatment, including a high-fibre diet, stimulants, and osmotic laxatives and enemas; (iv) absence of signs of megarectum or Hirschsprung’s disease on barium enema, or rectoanal inhibitory reflex on balloon distension of the rectum; (v) absence of small-bowel dysmotility, as measured by 6-hour GITT; (vi) absence of obstructed defecation syndrome, normal pelvic floor function and normal rectal compliance, as assessed by colpocystodefecography and anorectal manometry; (vii) strong patients’ desire to undergo the surgery; and (viii) absence of severe psychiatric disease. The choice between SC-ACRA and TC-IRA was mainly based on the consulting surgeon’s discretion. The diagnosis was confirmed by post-operative histopathological examination.

### Data collection

Patient information, including the age and sex of patients, period of constipation, defecation frequency before surgery, pre-operative CCFIS and GIQLI, and surgical and perioperative data such as operative time, intra-operative blood loss and length of post-operative hospital stay were obtained from the clinical database of the hospital. Data on surgical outcomes were collected via an interview of participants at the outpatient clinics. The surgical outcome data were evaluated, analysed and compared on the basis of CCFIS, GIQLI, CCIS, patients’ satisfaction and post-operative complications. We used the Clavien–Dindo classification of severity to compare post-operative complications [19].

### Surgical techniques

The TC-IRA and SC-ACRA procedures are outlined in detail in our previous reports [13]. All surgeries were performed by three surgeons who were experienced in both procedures. For the TC-IRA procedure, the incision of the peritoneum was initiated from the sacral promontory and was performed medially to laterally. After the inferior mesenteric pedicle was ligated and divided, the sigmoid colon and rectum were divided and mobilized. Thereafter, the descending colon and splenic flexure were mobilized. Further, the ileum, right colon and hepatic flexure, along with the proximal transverse colon, were mobilized, using a retroperitoneal approach with early ligation of the vascular pedicles and late division of the lateral attachments. Finally, we mobilized the transverse colon with the preservation of the omentum majus until the entire colon and terminal ileum had been mobilized. Sequentially, the division of the rectum was performed intra-corporeally using an endoscopic linear stapler and the terminal ileum division was performed extra-corporeally. Additionally, a laparoscopic intra-corporeal ileorectal anastomosis was performed to reconstruct the gastrointestinal tract. We performed pneumatic tests routinely.

For SC-ACRA, the main techniques and sequence of mobilization were the same as for TC-IRA. However, we severed all colonic vascular pedicles close to the bowel but carefully preserved the ileocolonic artery and its branches to the caecum. In addition, the ascending colon was divided 5–7 cm above the ileocaecal junction and the rectum was transected below the level of the sacral promontory. Moreover, we completed an appendectomy and an extracorporeal antiperistaltic cecoproctostomy was performed at the base of the caecum and rectal stump.

### Statistical analysis

Statistical analyses were performed using SPSS 19.0 for Windows (SPSS, Chicago, IL, USA). All groups of continuous variables were tested for normality in distribution using the Kolmogorov-Smirnov test. Normally distributed data are expressed as mean ± standard deviation (SD) and were compared using two-tailed Student’s *t*-tests. The Wilcoxon rank-sum test was used to compare continuous variables with skewed distributions, which are expressed as medians with ranges. Fisher’s exact or the chi-square test was used to compare categorical variables, Wilcoxon rank-sum test was used to test ranked data. All results with *P *<* *0.05 were considered statistically significant.

## Results

Between January 2005 and January 2015, 35 and 20 patients underwent TC-IRA and SC-ACRA, respectively. There were no significant differences in baseline characteristics between the two groups, e.g. age (*P *=* *0.655), sex (*P *=* *0.234), period of constipation (*P *=* *0.105), defecation frequency (*P *=* *0.698), CCFIS (*P *=* *0.696) and GIQLI (*P *=* *0.396) (**Tables 1****and****2**). No significant differences were noted either regarding operative time (*P *=* *0.096), intra-operative blood loss (*P *=* *0.286) or hospital stay (*P *=* *0.271) (**Table 1**).

**Table 1.** Patient demographics and operative variables

Characteristic	TC-IRA	SC-ACRA	*P*-value
	(*n *=* *35)	(*n *=* *20)	
Sex (male/female)	7/28	1/19	0.234
Age, years	45 (31–69)	43 (34–66)	0.655
Period of constipation, years	14 (4–31)	13 (5–38)	0.105
Operative time, minutes	122 ± 11	110 ± 17	0.096
Intra-operative blood loss, mL	20 ± 3	18 ± 2	0.286
Hospital stay, days	14.6 ± 2.3	12.5 ± 2.4	0.271

Continuous variables presented as mean ± standard deviation or median (range).

TC-IRA, total colectomy with ileorectal anastomosis; SC-ACRA, subtotal colectomy with antiperistaltic caecorectal anastomosis.

**Table 2.** Long-term outcomes of patients who underwent TC-IRA or SC-ACRA

Outcome	Pre-operation			During follow-up^a^			
	TC-IRA (*n* = 35)	SC-ACRA (*n* = 20)	*P*-value	TC-IRA (*n* = 35)	SC-ACRA (*n* = 20)	*P*-value	
Median defection frequency, times/day	1/7 (1/7–1/2)	1/7 (1/7–1/2)	0.698	3 (1/6–7)	3 (1/6–5)	0.578	
Median CCFIS	18 (13–22)	18 (13–23)	0.696	2 (0–20)^b^	2 (0–19)^b^	0.454
Median CCIS	–	–	–	0 (0–5)	0 (0–2)	0.333
Median GIQLI	81 (38–106)	80 (68–99)	0.396	122 (81–132)^b^	120 (80–132)^b^	0.661

^a^ The median follow-up is 72 months (range, 12–120 months).

^b^ Comparison between pre-operation and follow-up in each group, *P *<* *0.05.

TC-IRA, total colectomy with ileorectal anastomosis; SC-ACRA, subtotal colectomy with antiperistaltic caecorectal anastomosis; CCFIS, Cleveland Clinic Florida Constipation Score; CCIS, Cleveland Clinic Incontinence Score; GIQLI, Gastrointestinal Quality of Life Index.

### Long-term surgical outcomes

The median follow-up duration for all patients was 72 months (range, 12–120). No mortality occurred. During the follow-up period, no significant difference in defecation frequency was found between the two groups (*P *=* *0.578, **Table 2**). Similar results were found for CCFIS (*P *=* *0.454), CCIS (*P *=* *0.333) and GIQLI (*P *=* *0.661). However, significant improvement in CCFIS (both *P *<* *0.001) and GIQLI (both *P *<* *0.001) were noted between pre-operative levels and those during the follow-up in each group (**Table 2**).

The post-operative patient-satisfaction rates in the TC-IRA group were as follows: 31 cases (88.5%) with ‘very satisfied’, 2 (5.7%) with ‘satisfied’, 1 (2.9%) with ‘acceptable’ and 1 (2.9%) with ‘not satisfied’. Similar results were found in the SC-ACRA group with 18 cases (90.0%) of ‘very satisfied’, 1 (5%) of ‘satisfied’ and 1 (5%) of ‘not satisfied’. There were no significant differences in patients’ satisfaction rates between the two groups (*P *=* *1.000). Moreover, no significant difference in post-operative complication rates was found between the two groups (*P *=* *0.285) (**Table 3**).

**Table 3.** Post-operative complications during a median follow-up of 72 months

Complication	TC-IRA	SC-ACRA	*P*-value
	(*n *=* *35)	(*n *=* *20)	
Total	13 (37.1%)	5 (25.0%)	0.285
Small-bowel obstruction	2 (5.7%)	1 (5.0%)	1.000
Diarrhoea	3 (8.5%)	1 (5.0%)	1.000
Faecal leakage	2 (5.7%)	1 (5.0%)	1.000
Abdominal pain	4 (11.4%)	1 (5.0%)	0.643
Rectal pain	1 (2.8%)	1 (5.0%)	1.000
Incisional hernia	1 (2.8%)	0 (0.0%)	1.000
Clavien–Dindo classification			0.340
II	12 (34.2%)	5 (25%)	
III	1 (2.8%)	0 (0.0%)	

TC-IRA, total colectomy with ileorectal anastomosis; SC-ACRA, Subtotal colectomy with antiperistaltic caecorectal anastomosis.

## Discussion

STC is one of the common functional disorders encountered by colorectal surgeons currently. The primary treatment for STC comprises conservative medical methods such as dietary adjustments and the use of osmotic laxatives, etc. However, patients with intractable STC, whose symptoms of constipation do not improve after long-term non-operative treatments, should be considered as candidates for surgery [2, 20]. The current surgical procedures for STC include TC-IRA, subtotal colectomy with caecorectal anastomosis, subtotal colectomy with end-to-end antiperistaltic caecorectal anastomosis, subtotal colectomy with ileosigmoid anastomosis, colonic exclusion and antegrade intra-operative colonic lavage [2, 9, 21–24]. Of these, TC-IRA and subtotal colectomy with caecorectal anastomosis procedures are the main surgical options. However, no standard surgical procedure for STC has been established.

TC-IRA is considered to be an effective procedure and is recommended for first-line treatment of STC by many colorectal surgeons. Knowles *et al*. [25] showed that 47 of 59 (90%) patients with severe idiopathic STC were satisfied with the outcome after TC-IRA. Similarly, as Ripetti *et al.* [26] reported, almost all the patients with intractable STC had improvement of constipation. There were also statistically significant improvements in the SF-36 scores including those for physical pain, and the emotional, psychological and general health spheres, comparing before and after TC-IRA. However, there have been contradictory views. According to Di Fabio *et al.* [27], although most patients with intractable STC experienced improvement of constipation symptoms, they also had a significantly poorer post-operative quality of life in most of the physical, social and psychological domains. Moreover, Lim *et al.* [28] reported an incidence of faecal leakage of 20.4% and Hassan *et al.* [29] reported a high incidence of post-operative diarrhoea (approximately 30%) and small intestinal obstruction (10%). These post-operative complications strongly affected the patients’ quality of life and are also the reasons why subtotal colectomy with caecorectal anastomosis was invented.

Subtotal colectomy with caecorectal anastomosis was first proposed by *Lillehei* and *Wangensteen* [30], with an isoperistaltic end-to-end anastomosis between the rectal stump and caecum and a 180° rotation of the caecum from right to left to position the caecum in the left lilac fossa. Further, Deloyers *et al.* [31] and Zinzindohouè *et al.* [32] developed a modification of this technique with a craniocaudal rotation of the caecum in such a way that its anterior surface lies against the retroperitoneum of the right parietocolonic gutter and the cecal base points in a cephalad direction. Because all the above-mentioned subtotal colectomy procedures require a rotation of the caecum and the ileocolic vascular pedicle during surgery, they are difficult to perform, prolong the surgical time and might cause severe surgical complications. Thus, these procedures were not widely accepted or used for treating STC by most colorectal surgeons. Consequently, Sarli *et al.* [15] reported the use of SC-ACRA for the treatment of patients with intractable STC in 2001. Unlike the above-mentioned subtotal colectomy procedures, SC-ACRA not only preserves the caecum, ileocecal valve and terminal ileum to make a pouch to decrease the incidence of post-operative diarrhoea, but also does not require the rotation of the caecum and the ileocolic vascular pedicle and is simple to apply without major complications. In addition, the reported outcome was good, with a 100% improvement of constipation symptoms. A similar report by Iannelli *et al.* [33] demonstrated a high rate of success (79%) at a mean follow-up of 10.5 years, with only one case of diarrhoea and two cases of unchanged constipation. However, because of the small number of cases involved and lack of comparative studies on long-term outcomes between SC-ACRA and other procedures such as TC-IRA, the superiority of the SC-ACRA procedure needed further evaluation.

A longer operative time is considered to be a common disadvantage of subtotal colectomy with caecorectal anastomosis procedures because of their complexity. In the present study, we compared the perioperative characteristics of the TC-IRA and SC-ACRA groups, especially regarding operative time and intra-operative blood loss. In addition, our results showed no significant differences in the perioperative characteristics between both groups, indicating SC-ACRA was as simple as TC-IRA.

Post-operative complications such as faecal leakage and diarrhoea have been reported to strongly affect patients’ quality of life after TC-IRA [28, 29]. Subtotal colectomy with caecorectal anastomosis seemed to decrease these post-operative complications [15, 27–29]. Further, in the current investigation, we compared the post-operative long-term follow-up outcomes between the SC-ACRA and TC-IRA groups. However, no significant differences were found regarding the number of bowel movements, CCFIS and CCIS between the two groups. Similar results were also for GIQLI and patients’ satisfaction. In addition, we also analysed the incidence of post-operative complications including faecal leakage and diarrhoea and, similarly, no significant differences were found between the two groups. All the above-mentioned findings indicate that the long-term outcomes of SC-ACRA are similar to those of TC-IRA.

Although post-operative small-bowel obstruction (SBO) is one of the most commonly reported complications after total or subtotal colectomy [32], the incidences of post-operative SBO in the present study were 5.7% and 5.0% in the TC-IRA and SC-ACRA groups, respectively. These incidences are significantly less than previously reported levels of at least 18% [34, 35]. Abdominal pain and rectal pain persisted in 15.0% of the patients in the present study; however, their associated symptoms were gradually alleviated after surgery in both groups. In addition, no patient had symptoms of abdominal bloating and only one patient in each group had recurrent symptoms of STC in the current study.

This study has several limitations. First, it was a retrospective observational study. Second, the data of this study were from a single medical centre. Consequently, we will perform a prospective randomized–controlled trial with a long-term follow-up to further evaluate the findings in the present study.

In conclusion, both TC-IRA and SC-ACRA appeared effective for the surgical treatment of severe STC with similar long-term outcomes. However, the present study was a single-centre retrospective observational study and thus multicentre randomized–controlled trials should be conducted to validate these preliminary findings.

## Authors’ contribution

X.Y.X. contributed to data collections, data analysis and drafting the manuscript; K.L.S. contributed to writing the paper; W.H.C. contributed to data collections; Y.Z. contributed to data analysis; B.X.C. participated in data collections; Z.D. contributed to data collections; X.Q.Y. participated in the writing of the paper; Y.H.W. contributed to data analysis; Q.Q. participated in the writing of the paper; C.Q.J. contributed to study design, data collections, data analysis and writing the paper; W.C.L. contributed to study design, data analysis and writing the paper. All authors read and confirmed the final manuscript.

## Funding

This study was funded by grants from the National Natural Science Foundation of China [No. 81570492 to C.Q.J.] and the National Natural Science Foundation of China [No. 81500505 to W.C.L.] and the Natural Science Foundation of Hubei Province [No. 2015CFB636 to W.C.L.].
